# Graphene-Enhanced Methacrylated Alginate Gel Films for Sustainable Dye Removal in Water Purification

**DOI:** 10.3390/gels10010025

**Published:** 2023-12-27

**Authors:** Rubén Teijido, Qi Zhang, Miren Blanco, Leyre Pérez-Álvarez, Senentxu Lanceros-Méndez, José Luis Vilas-Vilela, Leire Ruiz-Rubio

**Affiliations:** 1 Macromolecular Chemistry Group (LQM), Physical Chemistry Department, Faculty of Science and Technology, University of the Basque Country (UPV/EHU), 48940 Leioa, Spain; ruben.teijido@bcmaterials.net (R.T.); leyre.perez@ehu.eus (L.P.-Á.); 2 BCMaterials, Basque Center for Materials, Applications and Nanostructures, UPV/EHU Science Park, 48940 Leioa, Spain; qi.zhang@bcmaterials.net (Q.Z.); senentxu.lanceros@bcmaterials.net (S.L.-M.); 3 Tekniker, Basque Research and Technology Alliance (BRTA), 20600 Eibar, Spain; miren.blanco@tekniker.es

**Keywords:** methacrylated alginate, hydrogels, films, graphene oxide, dye adsorption, water purification

## Abstract

Self-standing nanocomposite films were prepared by three-dimensional UV-induced radical copolymerization of methacrylated alginate (MALG) with acrylic acid (AA) and reinforced with graphene oxide (GO) to improve both mechanical strength and dye adsorption capacity in wastewater decontamination operations. Dynamic mechanical–thermal analysis revealed variations in storage modulus: the higher the GO content, the higher the storage modulus (E′) values. Also, the higher the temperature (associated with a lower and lower water content of films), the larger values of E′ for the films of the same composition (E′(25 °C) = 676.6–1538.7 MPa; E′(100 °C) = 886.9–2066.6 MPa), providing insights into the compatibility between GO and the MALG/AA matrix, as well as, assessing the improvement in the nanocomposite’s final mechanical properties. These crosslinked films in a dry state exhibited rapid water uptake and relatively short drying times (ca. 30 min at room temperature for the MALG/AA/GO composites) resulting from the swelling–drying studies and water contact angle measurements. The efficacy of methylene blue removal from water assessed via UV–VIS spectrometry revealed excellent results, expressed as an adsorption yield of 70–80% and 85–98% after 30 h and 258 h, respectively, of immersion time of films into an MB aqueous solution of 12.5 mg/L (as the contaminated water model). The reusability of the same films was evaluated by consecutive extraction processes of MB from the composite membranes when the content of desorbed dye was also spectrophotometrically monitored and conducted in acidic conditions (HCl aqueous solutions of pH 2). Overall, the introduction of GO in the developed self-standing MALG/AA nanocomposite films exhibited enhanced mechanical properties and increased efficiency for dye removal applications. Their great reutilization potential was highlighted by low drying times and a good ability to release the dye initially adsorbed. Thus, the prepared films could be suitable materials for sustainable and effective water treatment technologies.

## 1. Introduction

Polysaccharides stand out as biopolymers with considerable potential applications [1,2,3] based on their widespread availability, relatively low costs, and eco-friendliness. In recent years, there has been a growing interest in alginate, a naturally occurring substance abundantly found in the cell walls of numerous algae species, providing remarkable flexibility and toughness, enabling these algae to withstand the relentless forces of continuous waves and tidal activities [4]. Structurally, alginate is a linear copolymer of β-d-mannuronic acid (M) and its C-5 epimer α-l-guluronic acid (G) linked by ether 1→4 bonds. Each alginate species can be characterized by its M/G ratio, as it may greatly influence the material’s final mechanical characteristics [5,6]. The distinctive characteristics of alginate are related to its chemical structure, with an abundance of carboxyl and hydroxyl groups. This characteristic significantly increases their range of potential applications and allows a broad spectrum of modifications and physical blending with different materials to develop non-soluble 3D networks as a platform, giving form to novel bio-composites. Examples of applications include (a) the use of alginates as thermal barriers in highly energetic Li-ion batteries, blended with carboxymethyl cellulose and further crosslinked with Ca^2+^ ions [7]; (b) their ionotropic gelation with starch into nanobeads for drug delivery applications [8]; or (c) many possibilities for their different types of functionalization and compositional formulations in wastewater treatment applications [9].

When it comes to pollutant removal from wastewater, alginate-based materials might be a promising pH-sensitive adsorbent, effective in the adsorption of various species of hydrophilic and/or cationic aromatic compounds found in contaminated environmental water [10].

Organic dyes are some of the most important industrially produced pollutants in textile, paints, printing, cosmetics, and food processing and packaging industries, among others. If not controlled, these non-biodegradable substances may cause severe and long-term contamination in water streams. Methylene blue (MB) is one of the most significant dyes, taking into account its prevalence, and is found in various industries such as the textile for fabrics like cotton and wool and as a coloring agent in the production of paper, plastics, cosmetics, or pharmaceutical products, to highlight only a few examples [11]. MB’s main pernicious effects, when accumulated, range from blocking the sunlight and detaining the photosynthetic activity of aquatic species (cyanobacteria, microalgae, algae), to carcinogenic, mutagenic, and other highly toxic effects (eye burn, tissue necrosis) in animal species that live in or use water [12,13].

Different strategies have been commonly employed in the decontamination of organic dyes, including physicochemical methods such as precipitation, flocculation, electrodialysis, photocatalysis, or membrane separation [12,14]. However, adsorption methodologies have proven to be the most effective due to their high dye removal capabilities, application simplicity, compatibility with high water flows, and economic implementation [15,16]. Recently, polymeric membranes and, in particular, those fabricated from biopolymers (alginate, chitosan) have gained relevance over other materials, such as the considerably costly porous ceramics [17,18,19] due to their environmental friendliness, low cost, and availability. Polysaccharide modification, functionalization, or combination with other materials into high adsorption performance nanocomposites have lately gathered abundant research efforts [12,20,21]. Despite the high adsorption potential already evidenced by these biopolymers, such a property is strongly influenced by the chemical structure of the adsorbent, which in turn may be further modified in accordance with a pre-set purpose. As an example, the adsorption capability of ALG towards MB is mainly based on its abundant carboxylic groups [22], which are able to interact favorably with MB molecules and immobilize them onto the biopolymer network. Thus, chemically modifying ALG with a process that depletes the final structure from these functional groups could diminish its adsorption capacity regarding such a cationic dye.

This research work proposes a chemical functionalization of alginate with methacrylic groups by means of a methacrylic anhydride reaction with the hydroxyl groups in C-2 on every guluronic acid unit (G), thus leaving the carboxylic groups unaltered. Besides the chemical structure, variables such as mechanical strength and swelling/drying behavior, among others, need to be optimized for each specific application [21]. The methacrylic functionalities enable a UV-induced crosslinking process (in fact, a three-dimensional homopolymerization of MALG or a three-dimensional copolymerization of MALG and AA when AA was used), resulting in a hydrogel-like structure for the fabricated films.

Graphene oxide nanoplatelets could be excellent fillers in biopolymer-based nanocomposites for a large variety of applications. This inorganic 2D-layered material is well-known for its compatibility with biopolymers [23,24] due to the abundance of oxygen-bearing functionalities on platelet surfaces. These may favorably interact with most polar matrixes, thus reinforcing the global structure and providing additional adsorption sites in the GO sheets’ interlayered space. Based on the relatively high market price of GO, only two GO-containing formulations have been chosen, developed, and studied, MALG/AA/GO1.25 and MALG/AA/GO5, where 1.25 and 5 denote the percentages of GO with respect to the MALG component in the formulations aforementioned.

The different amounts of graphene oxide (GO) were incorporated into methacrylated alginate/acrylic acid (MALG/AA) films to evaluate their impact on the resulting mechanical properties and MB adsorption efficiency. The overall efficiencies and capacities were quantified through UV–VIS spectrophotometric analysis of aqueous MB solutions in which the fabricated films were immersed. In response to the demand for enhanced adsorption properties of materials concerning water pollutants, a study of the reutilization potential of the fabricated films was carried out involving the characterization of swelling–drying behavior and associated timeframes, as well as the evaluation of the extraction efficiency of adsorbed MB (as pollutant model in wastewater) under acidic conditions.

## 2. Results and Discussion

### 2.1. Synthesis and Characterization of Alginate and Its Methacrylated Derivative

**Figure 1:**
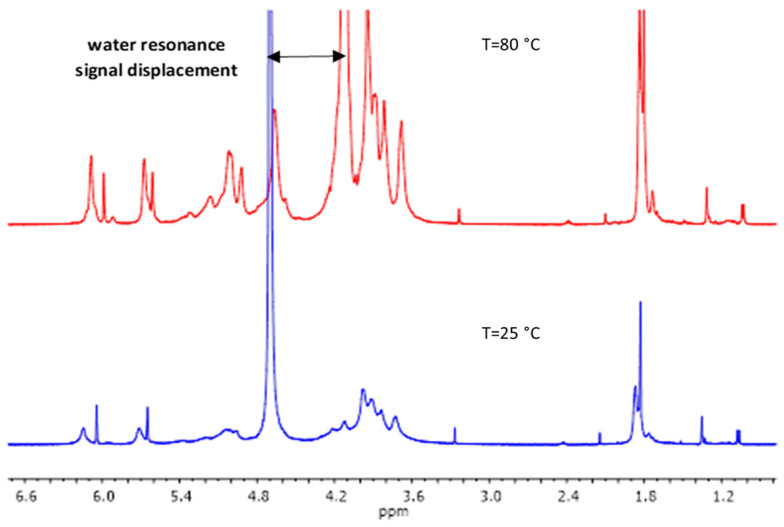
MALG ^1^H-NMR spectra showing the displacement of water resonance signal in the experiment performed at 80 °C.

The M/G ratio of the alginate influences the final mechanical properties of the materials [5,6]. Considering this, the M/G ratio of the used alginate was determined by ^1^H-NMR. The yield of the synthesis of methacrylated alginate was 78%. For the determination of the methacrylation degree (%MD), as suggested by Jensen et al. [25], MALG samples ^1^H-NMR spectra were acquired at an elevated temperature of 80 °C for two main reasons. Firstly, at 80 °C, the sample viscosity decreased, resulting in narrower signal widths. Secondly, the water resonance signal shifted out of the spectral region of interest (Figure 1), originally positioned between 4.5–5 ppm. This shift is very relevant as it resolves the overlap with signals from the anomeric protons in alginate G and M units, hindering the determination of %MD through these integration methodologies.

**Figure 2:**
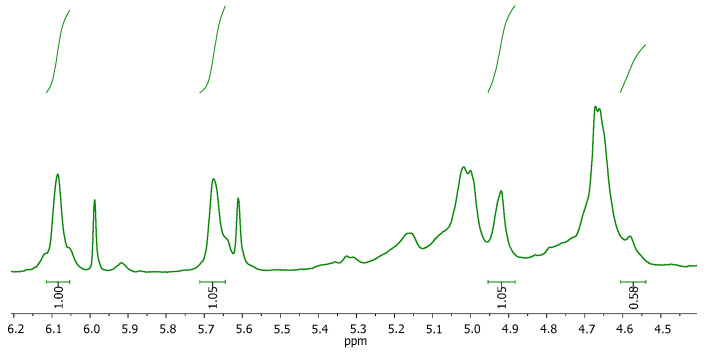
MALG ^1^H-NMR spectra with the integrated signals used in the characterization of the G and M units in MALG.

All calculations for the M/G ratio and %*MD* were carried out following equations 1 and 2 and considering the integration of the signals for the anomeric protons in *G* (*I_G_* = 4.9–5.0 ppm) and *M* (*I_M_* = 4.55–4.6 ppm) units, as well as the signals for vinylic protons in the methacrylated units (*I_A_* = 5.6–5.7, *I_B_* = 6.0–6.1 ppm) (Figure 2):

(1) $$\%G=\frac{I_{G}}{I_{M}+I_{G}}\times 100\%$$

(2) $$\%MD_{A}=\%G\times \frac{\frac{I_{A}+I_{B}}{2}}{I_{G}}\times 100\%$$

After analysing five samples, the proportion of *G* units was found to be 65 ± 1%, resulting in an M/G ratio of 0.539 and a %*MD* = 44.4 ± 2.9%.

### 2.2. Contact Angle Measurements

**Figure 3:**
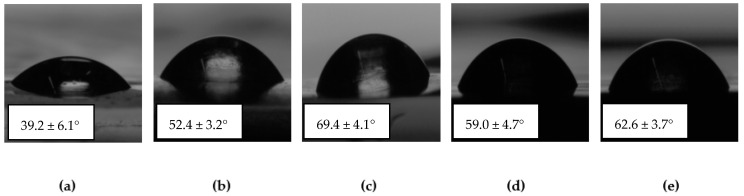
Values of the contact angle measured by the method of sessile water drops deposited on the film surfaces of (**a**) ALG, (**b**) MALG, (**c**) MALG/AA, (**d**) MALG/AA/GO1.25 and (**e**) MALG/AA/GO5.

The water wettability of the individual films is highly dependent on the surface roughness but also on their chemical functionalities and possible additives involved in their formulations. Water contact angle (WCA) results were obtained as the mean values of five measurements, and some representative images of sessile water drops are presented in Figure 3.

The water contact angle results evidenced the decrease in the hydrophilicity of these polysaccharide-based films (Figure 3) related to the GO increase in the formulation. The lowest value of WCA (39.2°) was obtained for pure alginate films. Furthermore, due to the poor physical crosslinking of this highly hydrophilic biopolymer, the sessile drop was rapidly absorbed by the film (local dissolution of alginate), leaving a hole on the surface. The stability problem of water drops was solved once chemical crosslinking was introduced into these systems. A first crosslinking approach consisted of three-dimensional radical homopolymerization of methacrylate alginate induced by UV-irradiation, which allowed to obtain higher WCA values (52.40°), but with the persistence of partial dissolution of the substrate on which the water drops were placed. On the other hand, by using a small amount of AA, the crosslinking process consisting of three-dimensional radical copolymerization of methacrylated alginate with AA most likely led to higher crosslinking density, with a direct consequence in increasing the value of contact angle up to 69.40° (the highest value). Instead, incorporating 1.25 wt.% of graphene oxide (GO) as a hydrophilic filler to enhance mechanical properties and dye adsorption capacity determined a reduction of water contact angle (WCAs) to 59°. At the same time, increasing GO content from 1.25 to 5% with respect to the MALG amount led to higher values of contact angle of 62.60°, due to the potential for physical crosslinking of these materials with the alginate-based matrix and the barrier effect of the impermeable GO nanoplatelets.

### 2.3. Mechanical Properties of MALG/AA and MALG/AA/GO Films

**Figure 4:**
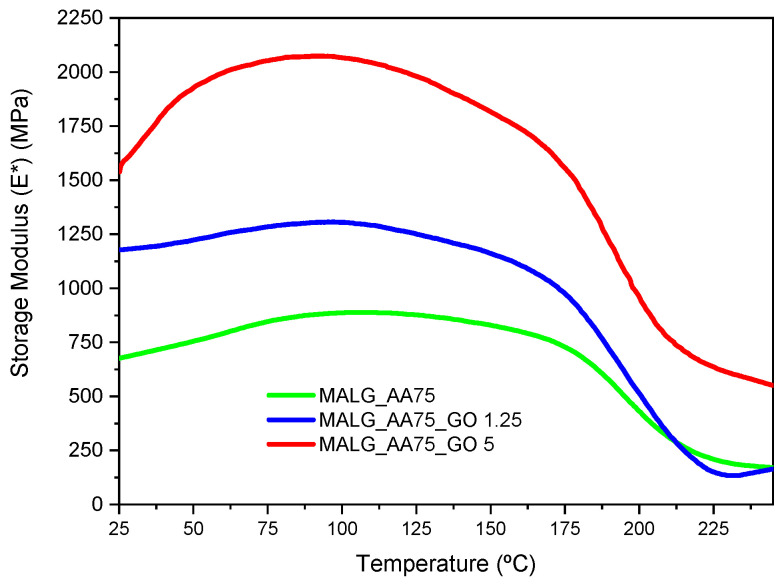
DMTA thermograms for the prepared MALG/AA films without and with the incorporation of different GO contents.

Dynamic mechanical thermal analysis (DMTA) was conducted on the self-standing films composed of MALG/AA, with various quantities of graphene oxide (GO) in their formulations. The results are presented in Figure 4, with the determined storage modulus values at two particular temperatures gathered in Table 1.

Due to the high hydrophilicity of these systems, it was noticed that the ambient humidity absorbed by the films before the DMTA experiments influenced their mechanical properties. This was evidenced by an increase in the storage modulus from 25 to 100 °C for all samples as the water content diminished. For this reason, two results of the storage modulus are presented, one at 25 °C and one at 100 °C. The latter values are considered indicative of completely dried films, reflecting the observed maximum storage modulus. It could be observed that the introduction of the fillers influenced the mechanical properties of the films. A substantial increase in the storage modulus was measured for film specimens bearing GO when compared to those without it. This effect was more evident at higher GO proportions, with values almost three times higher than those for the pristine polymer at the two presented temperatures. The high compatibility of GO functionality with the biopolymeric matrix facilitated more intimate filler-matrix interactions, thereby reinforcing the final structure of the material.

### 2.4. Dye Adsorption Onto MALG/AA and MALG/AA/GO Films

**Figure 5:**
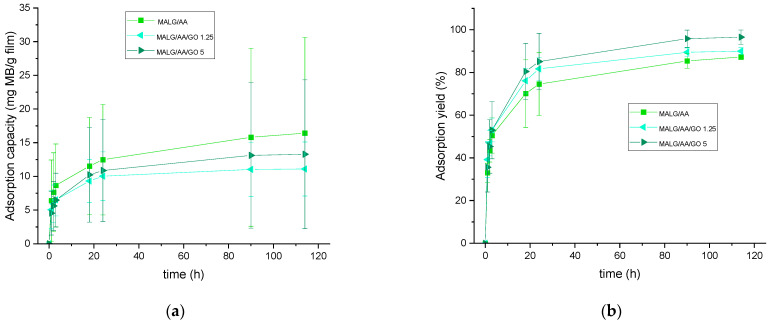
Methylene blue adsorption capacity (**a**) and overall adsorption yield (**b**) of the prepared MALG films as a function of time of exposure to MB aqueous solutions.

The potential applicability of these films for dye decontamination was monitored by UV–VIS spectroscopy. Thus, allowing for the calculation of the adsorption capacity per gram of material and the total amount of dye adsorbed or adsorption yield, the results are presented in Figure 5.

**Figure 6:**
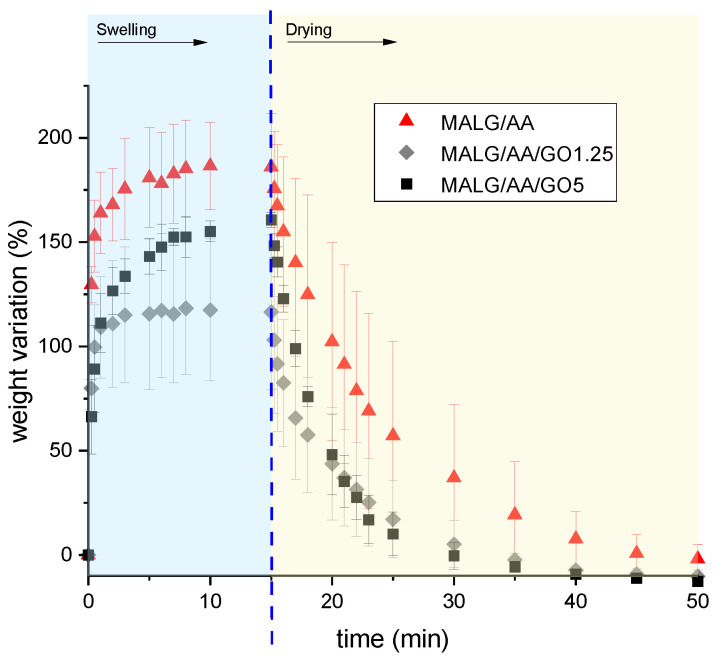
Weight variations during swelling–drying for the specified films.

**Figure 7:**
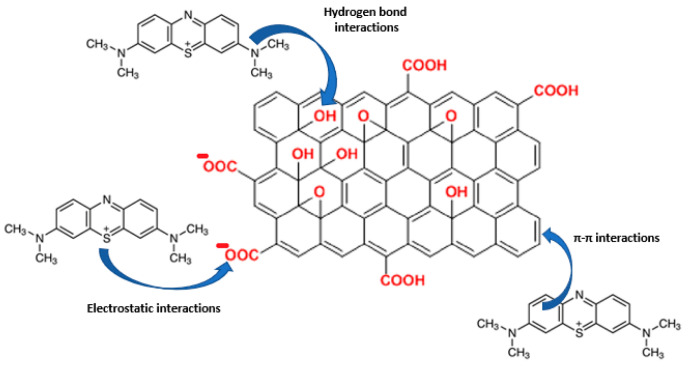
A possible adsorption mechanisms of GO for MB, a positive charge is allocated in the S atom, as it is one of the possible resonance forms of the MB molecule. (Red: Funtional groups present in GO).

The capacity for MB adsorption appeared to be related to the total water incorporation capacity of the fabricated films, following the same trend as in the swelling behavior (Figure 6). MALG/AA films showed higher capacities (17 mg MB/g film). In contrast, the capacities for the more intricate structures of MALG/AA/GO films were 10 mg MB/g film for 1.25 wt.% GO containing films and 12 mg MB/g film for films with 5 wt.% of GO, evidencing a compensation effect due to the MB molecules incorporated through interactions inbetween the GO layered structure. The apparent low reproducibility, as shown by large error bars (Figure 5a), was found to be most likely due to the significant influence of film thickness (or mass) exerted on this parameter. Thus, the reproducibility of the thickness of the films was low during the different sample fabrications due to the ad hoc molds used and the evaporation of the solvent during the crosslinking reaction. In other words, samples with different thicknesses produced a broad dispersion of adsorption capacity results, so that a better control over film thickness is expected to improve the consistency of measurements. However, the total efficiency for MB adsorption was increased in films with GO content from 88% of MALG/AA films to 90% on those bearing 1.25 wt.% of GO and 97% on those with a 5 wt.% of GO. Such an ascending tendency of MB adsorption with increasing GO content could be due to the great affinity of this dye to GO via multiple interaction types (hydrogen bonding, electrostatic interactions, π–π stacking), as suggested in Figure 7 [26].

For comparison and a better understanding of this process of MB adsorption, a brief collection of composites accompanied by specific mechanical properties and corresponding MB adsorption yields is listed in Table 2.

### 2.5. MALG/AA/GO Films Reusability

#### 2.5.1. Swelling–Drying Tests of the Prepared MALG Films

In addition, swelling–drying behavior was also evaluated, the results for each formulation being displayed in Figure 7.

The swelling–drying results evidenced good stability of the films, barely losing mass due to an almost negligible breaking or decomposition of the sample during the process. Additionally, the films presented a remarkable ability to spontaneously dry at ambient temperature, losing weight during the drying stage considerably faster, hinting at a good reusability in water-related applications. The incorporation of GO in the MALG/AA films had the effect of hindering the water adsorption, which led to the most substantial decrease of the equilibrium swelling for the films with 1.25% GO by ca. 33% compared to the equilibrium swelling of the films devoid of GO (Figure 7). This effect was slightly compensated by increasing the GO content to 5% and, as a result of the hydrophilic nature of the filler, the reduction in the equilibrium swelling of these systems compared to that of the films without GO was only 17%. During the drying stage, the films without GO were able to recover their initial weight in 30 min at ambient temperature in a ventilated room. Also, the same films showed shorter drying times, being 20 min for MALG/AA/GO1.25 films and only 15 min for MALG/AA/GO5 films. For this last case, the drying time of 15 min was equal to that characteristic of the entire swelling phase. The results revealed higher evaporation rates of water molecules physically bonded to the GO filler-containing films than those bonded to the alginate–acrylic acid matrix, the effect being more pronounced at an elevated filler content.

#### 2.5.2. Absorbed MB Extraction from MALG Films in Acidic Conditions

**Figure 8:**
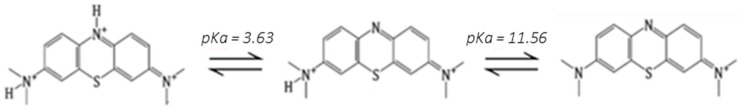
Acid-base equilibria of methylene blue.

To continue with the reusability characterization of the fabricated films, the possibility of extracting the absorbed MB was studied. MB bears ionizable tertiary amine functionalities in its chemical structure under acidic conditions (Figure 8) [30]. Thus, the selected experimental conditions for MB extraction include submerging the films in HCl solutions (0.01 M, pH = 2) for predefined periods of time when the dye positively charged is less and less adsorbed on an alginate matrix with an electrical charge increasingly close to zero in such an acidic medium, as reported by Othman et al. [31] and also employed by Fang et al. [32].

**Figure 9:**
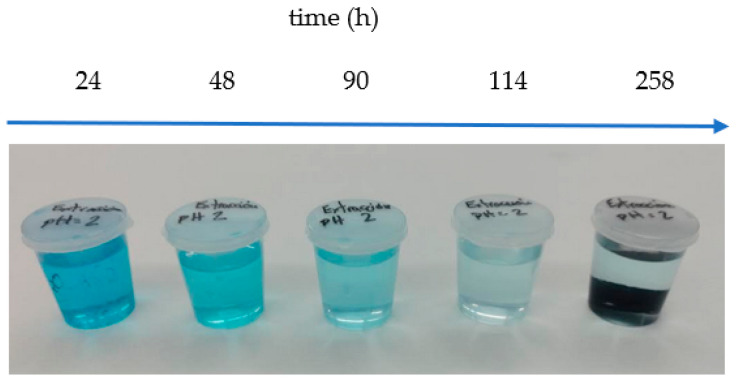
Image of the desorption solutions during the course of MB desorption from MALG/AA/GO5 films in HCl (0.01 M, pH = 2) aqueous solutions.

**Figure 10:**
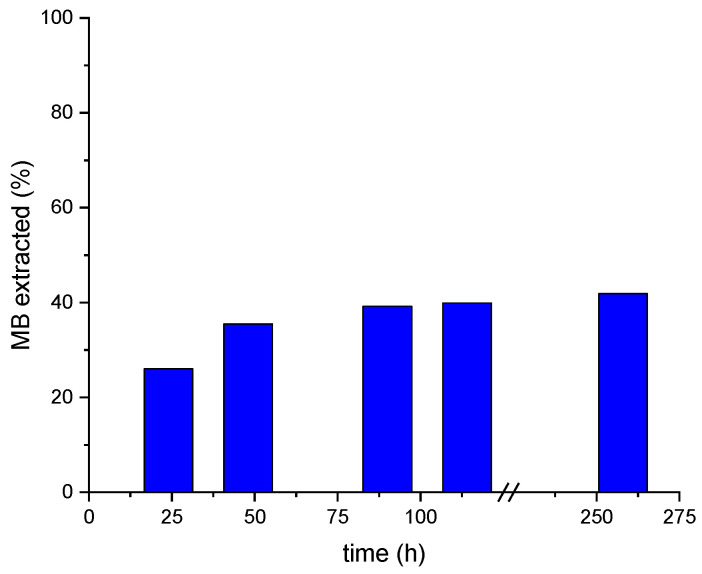
Accumulated MB extraction from a MALG/AA/GO5 used film during 258 h of its immersion in different HCl aqueous solutions of pH 2.

The MB desorption (assessed via UV–Vis spectrometry) was conducted under acidic conditions (Figure 9), determining the MB content in desorption solutions, namely, HCl aqueous solutions of pH 2, over a period of time of 258 h by using MALG/AA/GO5 films. This specific formulation was chosen due to its demonstrated higher adsorption efficiency within the same timeframe (Figure 5). These tests found good stability of the films in acidic media (pH = 2) during exposure time. However, as can be seen in Figure 10, only 42% of the total MB adsorbed by the MALG/AA/GO5 film was extracted over 258 h of immersion with a pronounced extraction during the first 24 h (ca. 26%), which indicates that a further improvement to these methodology needs to be achieved for a more efficient extraction of the initially adsorbed dye.

### 2.6. Additional Nanocomposite MALG/AA/GO Films Characterizations

Additional characterization techniques employed during the study of the fabricated films, such as cross-sectional SEM images and powder XRD diffractograms, are provided in the supporting information. The XRD diffractogram for a methacrylated alginate film (Figure S1) shows a characteristic highly amorphous signal for alginate with a maximum of around 26° with a shoulder to his left around 12–15°, corresponding to the GO maximum peak. This diffractogram is similar to others reported in the literature for alginate-based films containing GO [28]. Meanwhile, in the cross-sectional SEM images provided in Figure S2, the thickness of two film samples can be measured, resulting in 21 µm for pristine MALG film and 29 µm for a MALG film containing 5 wt.% of GO. Additionally, the platellets of GO, mostly oriented parallel to the film surfaces, can be observed in the images for the MALG/GO5 films.

## 3. Conclusions

This research work probed the possibility of easily fabricating alginate-based hydrogel-like films to treat dye-contaminated wastewater. The use of these adsorption technologies has already shown high efficiencies for dye removal, which, together with their composition based on abundant and inexpensive biopolymers, may produce new solutions that are affordable even for developing countries where these problems tend to be more acute. However, alginate films lack the mechanical properties to withstand their manipulation and integration in complex water treatment systems. Thus, this research showed how the inclusion of graphene oxide in alginate-based films can greatly improve their mechanical properties, leading to storage modulus values increased by a factor of 2.5. This improvement may allow, for example, the use of these self-standing films in applications with higher water flow rates. Furthermore, the affinity of GO for some dyeing products, such as methylene blue, that can be trapped onto/between GO layers, has the effect of greatly increasing their adsorption efficiency. Although some studies have been made for the potential reusability of these films, with good results for their drying times (35 min) once unsubmerged, improvements on the absorbed dye extraction strategy need to be achieved, as the treatment with acidic solutions proved not to be enough by extracting barely half of the amount of the absorbed dye.

## 4. Materials and Methods

### 4.1. Materials and Products

Sodium alginate (SA), methacrylic anhydride (MA, ≥94%), sodium hydroxide pellets (99%), glacial acetic acid (HAc), acrylic acid (AA), Irgacure-2959 photo-initiator and methylene blue (MB) were purchased from Sigma-Aldrich (St. Louis, MI, USA). Graphene oxide (GO) powder was provided by Graphenea (San Sebastian, Spain).

### 4.2. Methacrylation of Polysaccharides

Methacrylated alginate was synthesized through esterification of hydroxyl groups of alginate with MA. Briefly, a 2% *w*/*v* SA solution in deionized water was prepared and adjusted to pH = 8 using 5 M aqueous solution of NaOH. In the course of the first two hours of reaction time, MA at 20-fold excess was added slowly, while maintaining the temperature at 4 °C under vigorous stirring. During this time, pH was continuously monitored to be over a value of 8, adjusting when necessary by adding 5 M NaOH. Then, the solution was kept in a refrigerator for 22 h at 40 °C for the reaction to complete. Modified alginate was purified by dialysis (cellulose membranes, molecular weight cut-off −3.5 kDa, Medicell Membranes Ltd., London, UK) against 2 L of deionized water, with water periodically changed 8 times over 96 h to remove unreacted MA. By freeze-drying the dialyzed MA solution (−50 °C, 2.5 L Freeze Dryer, LAB-CONCO, Kansas City, MO, USA), the final product was obtained as a white sponge-like material with roughly 78% yielding.

### 4.3. Films Preparation

**Figure 11:**
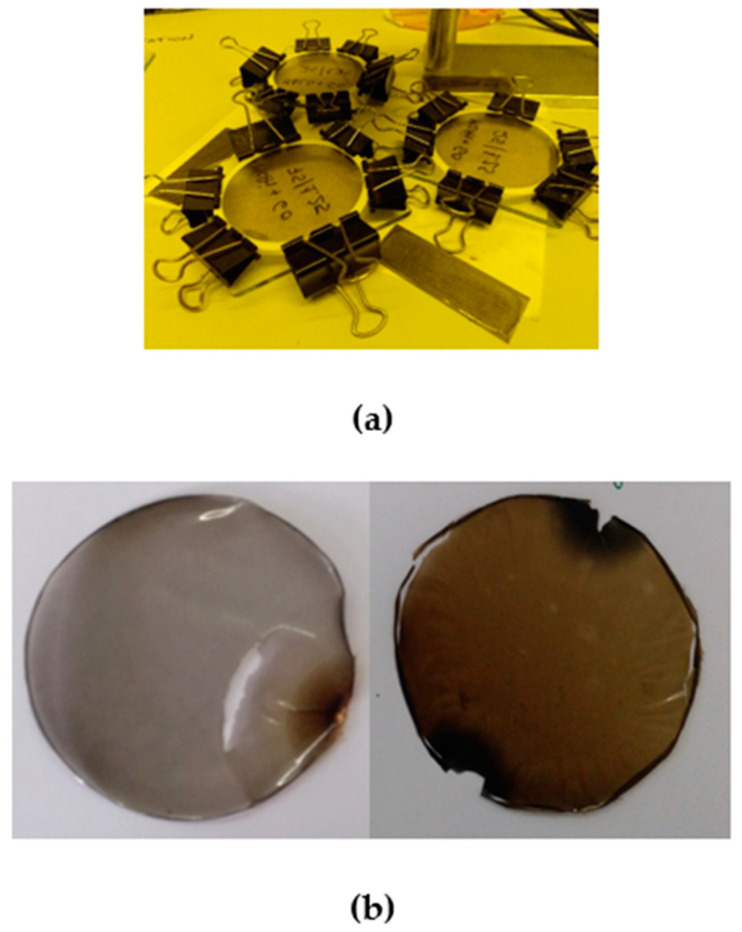
(**a**) Film curing process on the glass/Teflon molds and (**b**) two of the obtained self-standing film specimens.

Initially, MALG solutions (10 mL, 2% *w*/*v*) were prepared in deionized water. These solutions were mechanically stirred for 24 h and stored at 4 °C until further use. Meanwhile, aqueous dispersions of GO (1 mg/mL) were prepared, mechanically stirred (400 rpm, 15 min), and bath-sonicated (60 min) to ensure adequate dispersions. Before adding GO dispersions over polymer solutions, they were subjected to ultrasound processing (10 min, 40% amplitude, 20 kHz). For each film formulation preparation (MALG/AA75, MALG/AA75/GO1.25, and MALG/AA75/GO5), where 75 refers to the weight percentage of AA with respect to the MALG content), an appropriate amount of filler aqueous dispersion (0—2.5—10 mL) was added dropwise over the correspondent polysaccharide solution vigorous stirring to obtain certain weight percentages of GO with respect to the MALG amount (0, 1.25, and 5%, respectively). Then, a volume of 150 μL AA (AA75, 75 wt.% with respect to MALG amount) and 20 mg of the photo-initiator previously dissolved in 1 mL methanol were added onto every single MALG/GO aqueous dispersion under stirring. Finally, film-forming mixtures were introduced in a sonicating bath 10 additional minutes to ensure homogeneous dispersions of the components and eliminate bubbles that may produce defects during the curing process. The mixtures were then poured with the help of a syringe on the glass/Teflon molds built ad hoc (Figure 11).

Each mold was then placed under a UV lamp (65 W, 365 nm) for 30 min. After the UV exposure, the residual solvent was evaporated by introducing the molds into an oven (65 °C) until they were completely dried. Films were recovered by peeling them from the glass substrates after removing pincers and Teflon rings.

### 4.4. Alginate and Methacrylated Alginate Characterization

^1^H-NMR spectroscopy (500 MHz, D_2_O) was employed to determine the starting ALG mannuronic acid/guluronic residues ratio (M/G) as well as the modification degree (%MD) achieved during MALG synthesis (500 MHz, D_2_O, 80 °C) on a Bruker AVANCE 500 spectrometer (Billerica, MA, USA).

### 4.5. Water Contact Angle Measurements

These tests were conducted on an OCA 15EC device (DataPhysiscs Intruments, Filderstadt, Germany) able to perform contact angle measurements and drop shape analysis associated with the sessile drop method. Contact angle values were obtained by performing five measurements on average for different water drops (≈3 μL) placed on each film surface.

### 4.6. Dynamic Mechanical Analysis

The mechanical properties of the prepared self-standing films were studied by DMTA. DMTA thermograms in the tensile mode of each individual film (length: 5 mm, width: 6 ± 1 mm, thickness: 40 ± 10 µm) were recorded on a DMA 1 STAR System (Mettler Toledo, Columbus, OH, USA) instrument. The deformation amplitude was set to 3 μm, the frequency of the oscillatory deformation was 1 Hz, and the heating rate was 30 °C/min over the entire temperature range of 25–250 °C.

### 4.7. Dye Adsorption Experiment

In order to test the possible utilization of these films as dye adsorbents in water purification application, an aqueous solution of MB (250 mL, 12.5 mg/L) was prepared as a model of wastewater. Samples of the different self-standing films with a surface area of 5.1 ± 1.5 cm^2^, thickness of 45 ± 20 µm, and weight of 23 ± 10 mg were cut into smaller pieces and introduced in vials containing 20 mL of the prepared MB solution. After adsorption time, the remaining dye in each solution was quantified by UV–VIS spectroscopy. Each solution absorbance at 663 nm was monitored periodically (1, 2, 3, 18, 24, 90, 114, and 258 h) on a Cintra 303 UV–Visible Spectrometer (GBC Scientific Equipment Ltd., Melbourne, Australia), employing a corresponding standard curve for MB dissolved in distilled water.

### 4.8. MALG/AA Films Reutilization

The quantification of the extracted MB from MALG/AA/GO5 films in acidic conditions (by immersing them in HCl aqueous solutions of pH 2; these films were found to possess the highest adsorption efficiency over a period of time of 258 h and a good physico–mechanical stability under the same conditions) via UV–VIS spectrometry.

## Figures and Tables

**Table 1:** Storage modulus (E*, MPa) values of MALG/AA, MALG/AA/GO 1.25, and MCHI/AA/GO 5 at the specified temperatures.

Film Formulation	E* (MPa) at 25 °C	E* (MPa) at 100 °C
MALG/AA	676.6	886.9
MALG/AA/GO1.25	1178.2	1305.3
MALG/AA/GO5	1538.7	2066.6

**Table 2:** Published results on different mechanical properties and MB adsorption yields of some alginate-based systems.

Composite	Mechanical Properties	Adsorption Yield (%)	Ref.
Alginate/Ca^2+^/filter paper	Elastic modulus 773 (1 wt.% ALG) MPa 1907 (4 wt.% ALGSA) MPa	93.9	[27]
Alginate/Ca^2+^/graphene oxide	Not provided	91.3	[28]
Alginate/poly(acrylic acid)/TiO_2_ NPs	Storage modulus (G′) 971 MPa	80 (no TiO_2_NPs) 94.1 (0.05 g TiO_2_NPs) 99.4 (0.2 g TiO_2_NPs)	[29]
Methacrylated Alginate/poly(acrylic acid)/graphene oxide	Storage modulus (E′) 677 (no GO) MPa 1158 (1.25 wt.% GO) MPa 1539 (5 wt.% GO) MPa	88 (no GO) 90 (1.25 wt.% GO) 97 (5 wt.% GO)	Current work

## Data Availability

The data presented in this study are available upon request from the corresponding author.The data are not publicly available due to ethical.

## Supplementary Materials

The following supporting information can be downloaded at: https://www.mdpi.com/article/10.3390/gels10010025/s1. Figure S1: XRD powder diffractograms of the commercial GO and a MALG/GO5 film samples; Figure S2: Cross-sectional SEM images of (a), (b): MALG film at magnifications ×1500 and ×6000; (b), (c): MALG/GO5 film indicating the GO platelets at magnifications ×1500 and ×6000.

## References

[1] Costa J.A.V., Lucas B.F., Alvarenga A.G.P., Moreira J.B., de Morais M.G. (2021). Microalgae Polysaccharides: An Overview of Production, Characterization, and Potential Applications. Polysaccharides.

[2] Bilal M., Gul I., Basharat A., Qamar S.A. (2021). Polysaccharides-Based Bio-Nanostructures and Their Potential Food Applications. Int. J. Biol. Macromol..

[3] Yao Y., Xu B. (2022). Skin Health Promoting Effects of Natural Polysaccharides and Their Potential Application in the Cosmetic Industry. Polysaccharides.

[4] Venkatesan J., Nithya R., Sudha P.N., Kim S.K. (2014). Role of Alginate in Bone Tissue Engineering.

[5] Enobakhare B., Bader D.I., Lee D. (2006). Concentration and M/G Ratio Influence the Physiochemical and Mechanical Properties of Alginate Constructs for Tissue Engineering. J. Appl. Biomater. Biomech..

[6] Ramos P.E., Silva P., Alario M.M., Pastrana L.M., Teixeira J.A., Cerqueira M.A., Vicente A.A. (2018). Effect of Alginate Molecular Weight and M/G Ratio in Beads Properties Foreseeing the Protection of Probiotics. Food Hydrocoll..

[7] Bausch B., Frankl S., Becher D., Menz F., Baier T., Bauer M., Böse O., Hölzle M. (2023). Naturally-Derived Thermal Barrier Based on Fiber-Reinforced Hydrogel for the Prevention of Thermal Runaway Propagation in High-Energetic Lithium-Ion Battery Packs. J. Energy Storage.

[8] Thomas D., Mathew N., Nath M.S. (2021). Starch Modified Alginate Nanoparticles for Drug Delivery Application. Int. J. Biol. Macromol..

[9] Guo H., Qin Q., Chang J.S., Lee D.J. (2023). Modified Alginate Materials for Wastewater Treatment: Application Prospects. Bioresour. Technol..

[10] Zhang M.K., Zhang X.H., Han G.Z. (2022). Magnetic Alginate/PVA Hydrogel Microspheres with Selective Adsorption Performance for Aromatic Compounds. Sep. Purif. Technol..

[11] Pandey S., Ramontja J. (2016). Natural Bentonite Clay and Its Composites for Dye Removal: Current State and Future Potential. Am. J. Chem. Appl..

[12] Makhado E., Pandey S., Modibane K.D., Kang M., Hato M.J. (2020). Sequestration of Methylene Blue Dye Using Sodium Alginate Poly(Acrylic Acid)@ZnO Hydrogel Nanocomposite: Kinetic, Isotherm, and Thermodynamic Investigations. Int. J. Biol. Macromol..

[13] Zhang G., Yi L., Deng H., Sun P. (2014). Dyes Adsorption Using a Synthetic Carboxymethyl Cellulose-Acrylic Acid Adsorbent. J. Environ. Sci..

[14] Gong X.L., Lu H.Q., Li K., Li W. (2022). Effective Adsorption of Crystal Violet Dye on Sugarcane Bagasse–Bentonite/Sodium Alginate Composite Aerogel: Characterisation, Experiments, and Advanced Modelling. Sep. Purif. Technol..

[15] ALSamman M.T., Sánchez J. (2021). Recent Advances on Hydrogels Based on Chitosan and Alginate for the Adsorption of Dyes and Metal Ions from Water. Arab. J. Chem..

[16] Santander P., Butter B., Oyarce E., Yáñez M., Xiao L.P., Sánchez J. (2021). Lignin-Based Adsorbent Materials for Metal Ion Removal from Wastewater: A Review. Ind. Crops Prod..

[17] Singh V.P., Sharma M., Vaish R. (2019). Enhanced Dye Adsorption and Rapid Photo Catalysis in Candle Soot Coated Bi_2_WO_6_ ceramics. Eng. Res. Express.

[18] Singh V.P., Sharma M., Vaish R. (2020). Enhanced Dye Adsorption and Rapid Photocatalysis of Candle Soot Coated BaTiO_3_ Ceramics. Mater. Chem. Phys..

[19] Yadav J., Sahu O. (2023). Dye Removal of Cationic Dye from Aqueous Solution through Acid Functionalized Ceramic. Total Environ. Res. Themes.

[20] Al-Shemy M.T., Al-Sayed A., Dacrory S. (2022). Fabrication of Sodium Alginate/Graphene Oxide/Nanocrystalline Cellulose Scaffold for Methylene Blue Adsorption: Kinetics and Thermodynamics Study. Sep. Purif. Technol..

[21] ALSamman M.M., Sánchez J. (2022). Chitosan- and Alginate-Based Hydrogels for the Adsorption of Anionic and Cationic Dyes from Water. Polymers.

[22] Chen J.H., Li G.P., Liu Q.L., Ni J.C., Wu W.B., Lin J.M. (2010). Cr(III) Ionic Imprinted Polyvinyl Alcohol/Sodium Alginate (PVA/SA) Porous Composite Membranes for Selective Adsorption of Cr(III) Ions. Chem. Eng. J..

[23] Azizi-Lalabadi M., Jafari S.M. (2021). Bio-Nanocomposites of Graphene with Biopolymers; Fabrication, Properties, and Applications. Adv. Colloid Interface Sci..

[24] Rocha L.S., Nogueira J., Daniel-Da-Silva A.L., Marques P., Fateixa S., Pereira E., Trindade T. (2021). Water Softening Using Graphene Oxide/Biopolymer Hybrid Nanomaterials. J. Environ. Chem. Eng..

[25] Max Jensen H., Hofmann Larsen F., Balling Engelsen S., Stengel D.B., Conan S. (2015). Characterization of Alginates by Nuclear Magnetic Resonance (NMR) and Vibrational Spectroscopy (IR, NIR, Raman) in Combination with Chemometrics. Natural Products From Marine Algae: Methods and Protocols.

[26] Nissanka B., Kottegoda N., Jayasundara D.R. (2020). Probing Structural Variations of Graphene Oxide and Reduced Graphene Oxide Using Methylene Blue Adsorption Method. J. Mater. Sci..

[27] Qian L.W., Yang M.X., Zhang S.F., Hou C., Song W.Q., Yang J.F., Tang R.H. (2018). Preparation of a Sustainable Bioadsorbent by Modifying Filter Paper with Sodium Alginate, with Enhanced Mechanical Properties and Good Adsorption of Methylene Blue from Wastewaters. Cellulose.

[28] Ajeel S.J., Beddai A.A., Almohaisen A.M.N. (2021). Preparation of Alginate/Graphene Oxide Composite for Methylene Blue Removal. Mater. Today Proc..

[29] Thakur S., Pandey S., Arotiba O.A. (2016). Development of a Sodium Alginate-Based Organic/Inorganic Superabsorbent Composite Hydrogel for Adsorption of Methylene Blue. Carbohydr. Polym..

[30] Nishikiori H., Nagaya S., Tanaka N., Katsuki A., Fujii T. (1999). Acid-Base and Monomer-Dimer Equilibria of Methylene Blue in Dip-Coated Thin Films. Bull. Chem. Soc. Jpn..

[31] Othman I., Abu Haija M., Kannan P., Banat F. (2020). Adsorptive Removal of Methylene Blue from Water Using High-Performance Alginate-Based Beads. Water. Air. Soil Pollut..

[32] Fang Y., Liu Q., Zhu S. (2021). Selective Biosorption Mechanism of Methylene Blue by a Novel and Reusable Sugar Beet Pulp Cellulose/Sodium Alginate/Iron Hydroxide Composite Hydrogel. Int. J. Biol. Macromol..

