# Supplementary material for: Graphene-Enhanced Methacrylated Alginate Gel Films for Sustainable Dye Removal in Water Purification
Source: Gels. 2023 Dec 27;10(1):25. doi: 10.3390/gels10010025 (PMC10815123; doi:10.3390/gels10010025)
Supplement: Supplementary file 1 [file gels-10-00025-s001.zip › gels-2756882-supplementary.pdf]

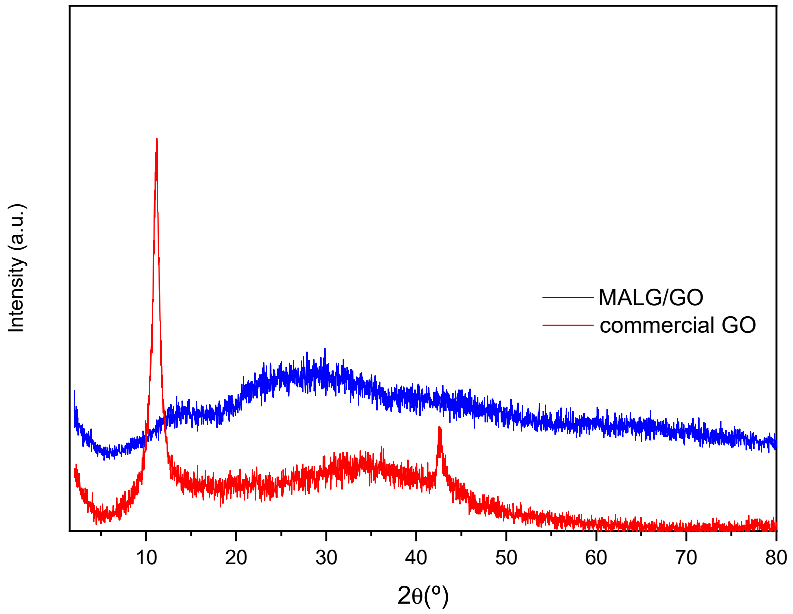

Figure S1. XRD powder diffractograms of the commercial GO and a MALG/GO5 film samples.

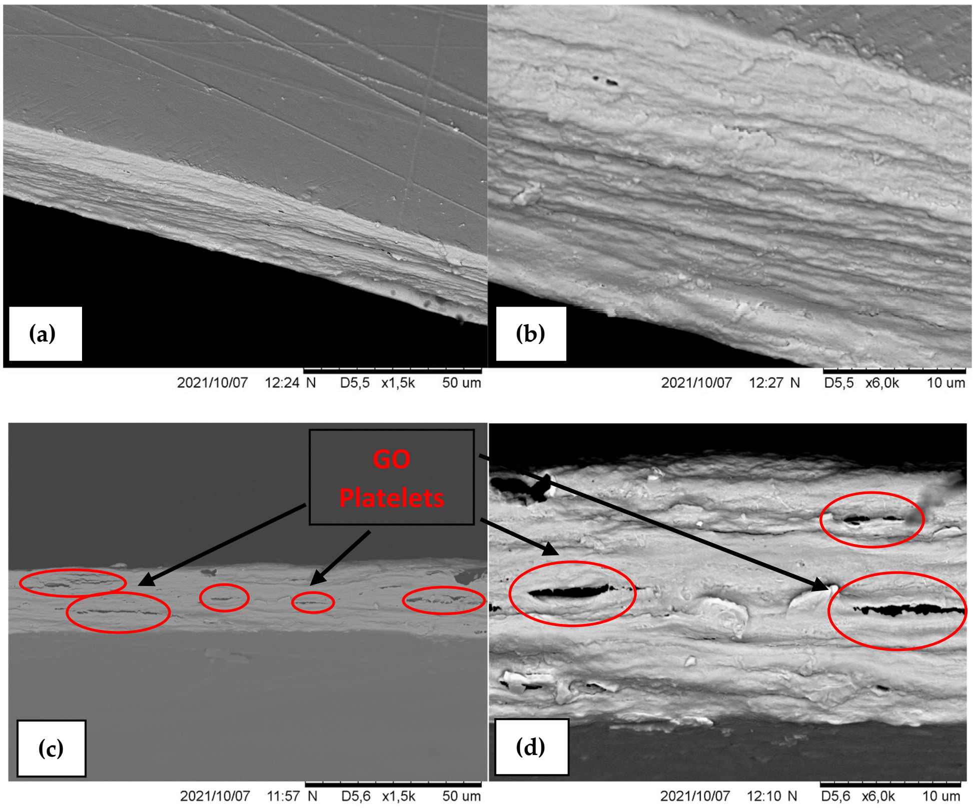

Figure S2. Cross-sectional SEM images of (a), (b): MALG film at magnifications x1500 and x6000; (b), (c): MALG/GO5 film indicating the GO platelets at magnifications x1500 and x6000.
